# Full-length transcriptome sequencing analysis and characterization, development and validation of microsatellite markers in *Kengyilia melanthera*

**DOI:** 10.3389/fpls.2022.959042

**Published:** 2022-07-25

**Authors:** Yanli Xiong, Jian Yang, Yi Xiong, Junming Zhao, Lin Liu, Wei Liu, Lina Sha, Jiqiong Zhou, Minghong You, Daxu Li, Xiong Lei, Shiqie Bai, Xiao Ma

**Affiliations:** ^1^College of Grassland Science and Technology, Sichuan Agricultural University, Chengdu, China; ^2^Sichuan Academy of Grassland Science, Chengdu, China

**Keywords:** *Kengyilia melanthera*, full-length transcriptome, SMRT sequencing, SSRs development, transferability

## Abstract

As a typical psammophyte of the Triticeae, *Kengyilia melanthera* possesses high feeding potential and great utilization values in desertification control in the Qinghai-Tibet Plateau. However, few gene function and genetic studies have been performed in *K. melanthera*. In this study, single-molecule real-time sequencing technology was used to obtain the full-length transcriptome sequence of *K. melanthera*, following the functional annotation of transcripts and prediction of coding sequences (CDSs), transcription factors (TFs), and long noncoding RNA (lncRNA) sequences. Meanwhile, a total of 42,433 SSR loci were detected, with 5′-UTRs having the most SSR loci and trinucleotide being the most abundant type. In total, 108,399 SSR markers were designed, and 300 SSR markers were randomly selected for diversity verification of *K. melanthera*. A total of 49 polymorphic SSR markers were used to construct the genetic relationships of 56 *K. melanthera* accessions, among which 21 SSR markers showed good cross-species transferability among the related species. In conclusion, the full-length transcriptome sequence of the *K. melanthera* will assist gene prediction and promote molecular biology and genomics research, and the polymorphic SSR markers will promote molecular-assisted breeding and related research of *K. melanthera* and its relatives.

## Background

The *Kengyilia* is a perennial genus in the tribe Triticeae of Poaceae with the genome constitution of StStYYPP (2*n* = 6 × = 42; Yen and Yang, 2020). It is only distributed in the temperate zone of the northern hemisphere with the distribution range roughly between 29° to 48° N and 57° to 104° E (Bing, 2001). The genus *Kengyilia* can provide important genetic resources for forage breeding and cereal improvement owing to its high feeding values and excellent resistance to biotic and abiotic stresses (Yen and Yang, 2020). As a typical psammophyte species of *Kengyilia* genus, *Kengyilia melanthera* mainly distributed in the Qinghai-Tibet Plateau (QTP) areas at an altitude of 3,300–4,750 m and possessed great utilization potential in desertification control and ecological restoration (Yen and Yang, 2020). Furthermore, the strong drought resistance and high carbohydrate content of *K. melanthera* make it suitable for artificial grassland building in QTP areas to cope with the seasonal shortage of forage.

*Kengyilia* is a new genus established in 1990 (Yen and Yang, 1990). Therefore, more attention has been paid to the genetic diversity and taxonomy of *Kengyilia* species in previous studies. The application of gene sequences in genetic diversity detection of *Kengyilia* including nuclear genes, namely, *Pgk1* (Fan et al., 2012) and *DMC1* (Gao et al., 2016), and chloroplast genes, namely, *trnL-F* (Zhang et al., 2009), *trnT-trnL* (Gao et al., 2014), and *matK* (Luo et al., 2012), was reported. Considering the simplicity, polymorphism, and stability, some molecular markers (Zhou et al., 2000; Zhang et al., 2005, 2008) have been applied to the phylogenetic relationship analysis of *Kengyilia*. Compared to those markers, SSR markers have greater advantages including their easy development, codominance, and high polymorphism (Wu et al., 2020).

Compared to G-SSR (genomic SSR), EST-SSR (expressed sequence tag SSR) possesses higher cross-species transferability and the ability to mark functional genes (Karcι et al., 2020). Recently, the increasing utilization of transcriptome sequencing makes it an efficient and cost-effective tool for EST-SSR marker development. Although there have been many reports about SSR identification and development *via* second-generation transcriptomic sequencing, little research is concerned with the SSR development using full-length transcriptome sequencing technology (Ge et al., 2019; Wu et al., 2020). Compared to the second-generation transcriptome sequencing, the SMRT sequencing technology hold more advantages including longer read length, less sequencing, and assembly error, which is more beneficial for the study of plants without a reference genome (Thomas et al., 2014; Gordon et al., 2015). The acquisition of full-length transcripts contributed to the gene annotation and identification of isoforms, fusion transcripts, and long noncoding RNA (lncRNA) (Zhao et al., 2019). Therefore, more SSRs can be discovered, and complete functional genes can be explored in this case.

In this study, first, the full-length transcriptome of the *K. melanthera* was obtained, and the functional annotation was performed to better understand its functional classification. Second, we characterized the SSRs of the *K. melanthera* by analyzing the frequency, distribution, and function of SSRs in the transcriptome. Finally, newly developed EST-SSR markers were used for genetic diversity and structure study in 56 *K. melanthera* accessions and other *Kengyilia* populations.

## Materials and methods

### Plant material collection and DNA and RNA extraction

The *K. melanthera* was collected from the nursery base of Sichuan Academy of Grassland Sciences (Chengdu, China) in Qiongxi Town, Hongyuan County (32°48′N, 102°33′E) (Supplementary Figure 1). To obtain as many transcripts as possible, the roots, stems, leaves, and inflorescences of a single plant were collected and stored in liquid nitrogen rapidly. A total of mixed 2 g sample containing the equal amounts of each tissue was used for RNA extraction and full-length transcription sequencing. The wild germplasms used to identify the polymorphism of the developed SSRs were collected from the nursery base of Sichuan Academy of Grassland Sciences in Hongyuan County, the sandy land in Waqie town (33°10′N, 102°37′E) and the sandy land in Dazhasi town (33°40′N, 102°56′E). The young leaves were stored in silica gel (Supplementary Table 1). The seeds of a further four related species, *Kengyilia alatavica*, *Kengyilia batalinii*, *Kengyilia mutica*, and *Kengyilia rigidula*, all came from the U.S. National Plant Germplasm System and were planted in the greenhouse of Sichuan Agricultural University (Supplementary Table 2). Total DNA and RNA were extracted by DNA extraction kit and RNA extraction kit (Tiangen Biotech, Beijing, China), respectively. Their concentration and quality were checked using NanoDrop1 ND-1000 Spectrophotometer (NanoDrop Technologies, United States) and agarose gel electrophoresis, respectively.

### PacBio library construction and sequencing

The qualified mixed RNA samples of roots, stems, leaves, and inflorescences were used to construct cDNA library. SMARTer PCR cDNA Synthesis Kit (Clontech, Mountain View, CA, United States) was used to synthesize cDNA, and then PCR amplification, quality control, and purification were performed. The 1–6 kb cDNA fragments were generated using BluePippin Size Selection System (Sage Science, United States). The SMRT sequencing libraries was constructed using the Pacific Biosciences DNA Template Prep Kit 2.0. Qubit 2.0 and Agilent 2100 were used to detect the concentration and quality of cDNA libraries, respectively. Finally, SMRT sequencing was performed on the PacBio RS II platform (Pacific Biosciences, Menlo Park, CA, United States).

### Quality filtering and error correction of PacBio long reads

Raw reads were processed into circular consensus sequences (CCSs) according to the adaptor, with full pass ≥3 and sequence accuracy >0.9. Next, full-length, non-chimeric transcripts were detected by searching the polyA tail signal and the 5′ and 3′ cDNA primer sequences in CCSs. The IsoSeq module in SMRT Link version 5.0.1 software (Pacific Biosciences of California, Inc., Menlo Park, CA, United States) was used to group the full-length sequences of the same transcript, and the similar full-length sequences were grouped into a cluster. Each cluster contained a consistent sequence, and the corrected consistent sequences were used to obtain high-quality sequences (accuracy >99%) for subsequent analysis. After removing the low-quality and high-quality redundant sequences (identity >0.99) using CD-HIT software (Li and Godzik, 2006), non-redundant high-quality transcripts were obtained.

### Functional annotation

The obtained transcript sequences were aligned to the NCBI non-redundant protein sequences (NR) (Deng et al., 2006), Protein family (Pfam) (Finn et al., 2014), Clusters of Orthologous Groups of proteins (COG) (Tatusov et al., 2000), euKaryotic Ortholog Groups (KOG) (Koonin et al., 2004), Evolutionary Genealogy of Genes: Non-supervised Orthologous Groups (eggNOG), Kyoto Encyclopedia of Genes and Genomes (KEGG) (Minoru et al., 2004), Gene Ontology (GO) (Ashburner et al., 2000) using BLAST v2.2.26 (Altschul et al., 1997) (*E*-value < 10-5).

### Predictions of coding sequences, transcription factors, and long noncoding RNA

TransDecoder v3.0.0^1^ was used to identify coding sequences (CDSs) based on Pfam database. ITAK v1.2 (Yi et al., 2016) was used to identify transcription factors (TFs), transcription regulators (TRs), and protein kinases (PKs).

Coding Potential Calculator (CPC) (Kong et al., 2007), Coding-Non-Coding Index (CNCI), Coding Potential Assessment Tool (CPAT) (Wang et al., 2013), and Pfam database were used to screen nonprotein-coding RNA candidates. The lncRNA candidates were predicted with the following criterion: the transcripts longer than 200 nt and possessing more than two exons. The predicted lncRNA candidates were further screened in CPC/CNCI/CPAT/Pfam.

### SSRs identification and primer design

Transcripts with the length more than 500 bp were used to identify SSRs based on MISA (Beier et al., 2017) software. The SSR loci were identified with the following criteria: repeat numbers of mono-, di-, tri-, tetra-, penta-, and hexa-nucleotide motifs greater than or equal to 10, 6, 5, 5, 5, and 5, respectively. Finally, Primer3.0 (Untergasser et al., 2012) was used to design primers with the primer size of 18–25 bp, annealing temperature of 55–65°C, GC content of 30–70%, and product length of 100–300 bp.

### Validation and evaluation of SSR markers

A total of 300 SSR primer pairs were randomly selected for synthesis and further validation. The PCR system included 10 μl of 2× Taq Master Mix (Tiangen Biotech, Beijing, China), 1 μl of forward/reverse primer (20 ng/μl), 2 μl of genomic DNA (20 ng/μl), and 6 μl of ddH_2_O. PCR was performed on Biometra Tadvanced under the following process: 94°C for 5 min, followed by 35 cycles including 30 s at 94°C, 30 s at 56 or 58°C, and 30 s at 72°C, and then extension at 72°C for 5 min.

Given that the heterohexaploid feature of *K. melanthera*, it is difficult to record the allelic variation. Therefore, the amplified bands were recorded as the 0/1 (presence/absence of bands) binary matrix. GenAlEx 6.503 (Peakall and Smouse, 2010) was used to calculate the number of alleles (Na), number of effective alleles (Ne), and Shannon’s information index (I). The polymorphic information (PIC) and expected heterozygosity (He) were calculated based on the formula of PIC = 1 − *p*^2^ − *q*^2^, where *p* and *q* are frequency of present/absent band; He = 1 − Σ*pi*^2^, where *pi* is frequency of the *i*-th allele (Zhang et al., 2019; Zheng et al., 2020). The Nei genetic distance (GD) matrix was obtained by Freetree (Hampl et al., 2001), the UPGMA (unweighted pair-group method with arithmetic means) dendrogram was constructed, and the visualization was performed on Figtree (Hampl et al., 2001). Population structure was speculated using STRUCTURE v2.3.4 (Falush et al., 2007) with 50,000 burn-in and 100,000 Monte Carlo Markov chain (MCMC). Then the optimal *K* value was determined using STRUCTURE HARVESTER (Earl and Vonholdt, 2012). The principal coordinate analysis (PCoA) was carried out by NTSYS v2.2 (Rohlf, 1987).

## Results

### General properties and functional annotations of full-length transcriptome

**FIGURE 1:**
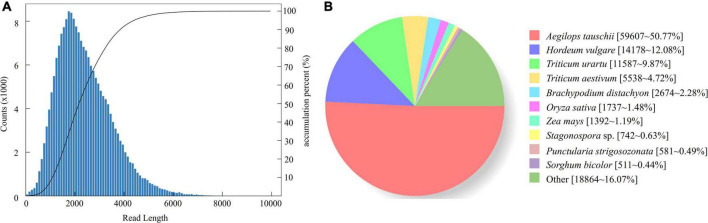
Length distribution of 204,028 consensus isoforms in *K. melanthera* **(A)** and homologous species distribution of *K. melanthera* based on the NCBI non-redundant protein sequences (NR) database **(B)**.

A total of 96.1 GB raw data was obtained (GenBank database accession number: PRJNA735213). There were 542,441 CCSs (read bases of 1,399,665,069) with an average length of 2,397 bp and mean number of passes of 35. We obtained 491,001 full-length non-chimeric (FLNC) reads, and the percentage of FLNC is 90.52%. After clustering the FLNC sequences, we obtained 4,580 polished low-quality isoforms and 199,134 (97.60%) polished high-quality isoforms. Finally, 204,028 consensus isoforms were obtained, with the average length of 2,399 bp (Figure 1A). After removing the low-quality and redundant high-quality transcripts, 126,410 transcripts were obtained. In order to access the completeness and accuracy of transcripts obtained in this study, we also aligned the transcripts to OrthoBD database. The results showed that 82.57% (1,189 of 1,440) of transcripts were completed with only 4.52 and 12.91% being fragmented and missing.

**FIGURE 2:**
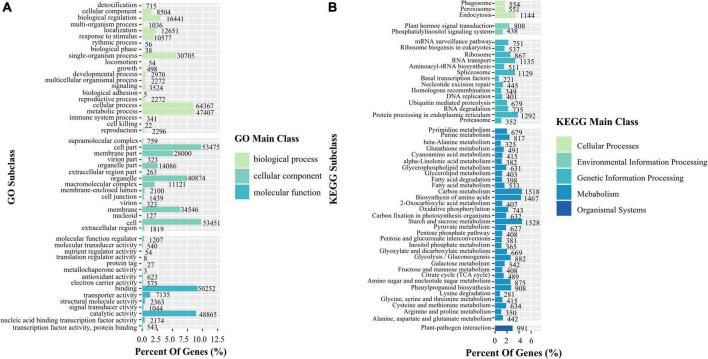
Functional classification of transcripts based on the GO **(A)** and KEGG **(B)** in *K. melanthera*.

A total of 118,341 transcripts of *K. melanthera* were perfectly matched with the COG, GO, KEGG, KOG, Pfam Annotation, Swissprot Annotation, eggNOG, and Nr databases (Supplementary Table 3). Sequence alignment based on NR database showed that three species with the highest homology with *K. melanthera* all belonged to the Triticeae (Figure 1B), among which *Aegilops tauschii* (50.77% of transcripts) had the highest homology with *K. melanthera*, followed by *Hordeum vulgare* (12.08% of transcripts) and *Triticum urartu* (9.87% of transcripts). A total of 91,155 and 44,928 transcripts were assigned into 51 subcategories of three major GO functional categories and 130 KEGG pathways (Figures 2A,B), respectively. The pathways involving the largest number of transcripts were *starch and sucrose metabolism* (1,528), *carbon metabolism* (1,518), and *biosynthesis of amino acids* (1,467).

In this study, a total of 48,095, 110,413, and 69,144 transcripts were assigned to 25 COG categories, 23 eggNOG categories, and 25 KOG categories. The largest number of transcripts were annotated in the *signal transduction mechanism* category (5,893) in COG categories (Supplementary Figure 2a). Among the 23 eggNOG categories, in addition to *function unknown*, most of the transcripts (8,403) were annotated with the *signal transduction mechanism* category (Supplementary Figure 2b). In KOG categories, most transcripts were assigned to the *general function prediction only* category (12,904), followed by the *signal transduction mechanism* (10,135) (Supplementary Figure 2c).

### Analysis of coding sequences, transcription factors, and long noncoding RNAs

**FIGURE 3:**
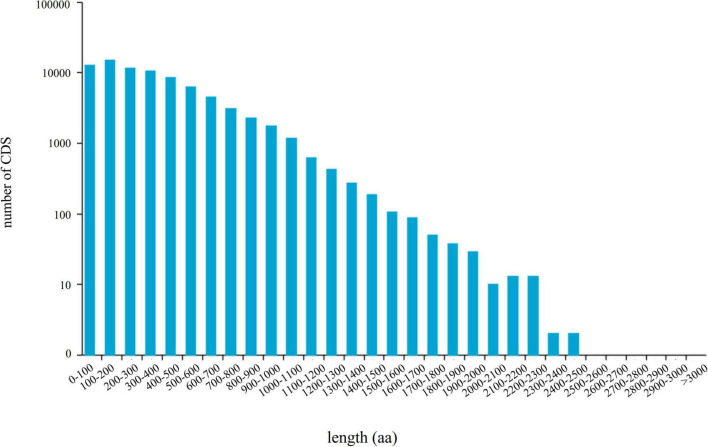
The length distribution of 79,159 CDS-encoded protein sequences.

**FIGURE 4:**
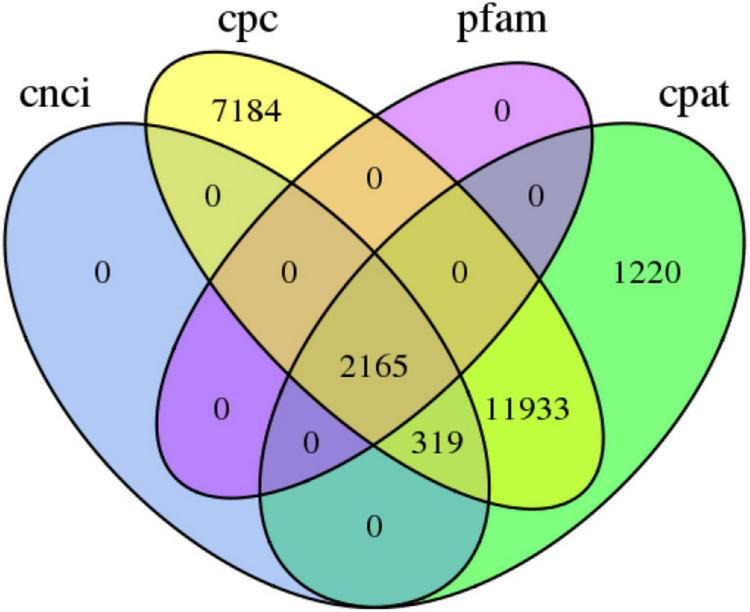
The number of long noncoding RNA transcripts predicted according to the CPC, CNCI, CPAT, and Pfam databases.

A total of 79,159 CDSs (Figure 3) and 12,355 TFs belonging to 222 families were identified (Supplementary Table 4). The most abundant TF family was *RLK-Pelle_DLSV* (1,122), followed by *C2H2* (412) and *RLK-Pelle_LRR-XII-1* (282). Four databases CNCI, CPC, Pfam, and CPAT were used to predict the lncRNAs. The results showed that there were 2,484, 21,601, 2,165, and 15,637 lncRNA candidates with length ≥200 bp and exon ≥2. Finally, 2,165 transcripts shared in four databases were considered as the potential lncRNA (Figure 4).

### Characterization of SSRs in transcriptome

A total of 42,433 SSRs (Table 1) were identified from 31,862 transcripts in the *K. melanthera* transcriptome. The frequency of SSRs was 35.87%, and an average of one SSRs loci was found every 7.19 kb. The most abundant repeat motif type was trinucleotide (45.1%), followed by mononucleotide (30.1%) and dinucleotide (21.0%) (Supplementary Figure 3), while pentanucleotide and hexanucleotide accounted only for 0.6 and 0.5%. A/T (83.74%), AG/CT (54.75%), CCG/CGG (31.09%), ACAT/ATGT (11.28%), AGAGG/CCTCT (12.41%), and ACCGCC/CGGTGG (5.56%) were the most abundant motifs in mono- to hexanucleotide repeats, respectively (Supplementary Figure 3).

**TABLE 1:** General information for the SSR analysis.

Items	Number
SSR number detected	42,433
Number of sequences containing SSR	31,862
Number of sequences containing ≥1 SSR	7,535
Number of compound SSRs	3,083
Frequency of SSRs loci (%)	35.87
Distribution density of SSRs loci (kb)	7.19

**FIGURE 5:**
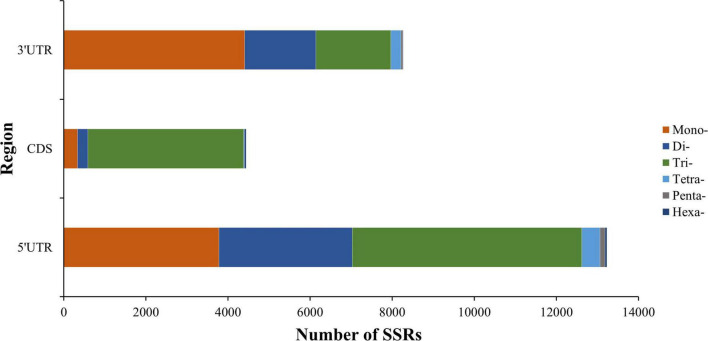
Distribution of six SSR repeat types in different genic regions.

We also explored the position distribution feature of SSRs in *K. melanthera* transcriptome (Figure 5). A total of 25,944 SSRs were identified in the CDS and untranslated regions (UTRs), of which the 5′-UTR region had the most SSRs (13,234), followed by the 3′-UTR (8,270) and the CDS region (4,440). Totally, trinucleotide (85.4%), mononucleotide (53.3%), and dinucleotide SSRs (42.3%) were the most repeat types in the CDS, 3′-UTRs and 5′-UTRs regions.

In total, 8,458 transcripts holding SSRs were categorized into three functional categories (Supplementary Figure 4a). *Metabolic process* (5,227) and *cellular process* (4,285) were the two subcategories with the most transcripts in the “biological process category.” In the “cellular component,” *cell* (2,319) and *cell part* (2,322) are the two subcategories that involved the greatest number of transcripts. The two subcategories of *catalytic activity* (4,541) and *binding* (4,938) involved the most transcripts in the “molecular function category.” In addition, based on KEGG pathway analysis, 8,833 transcripts were assigned to 124 pathways. The three most abundant transcript pathways are *Carbohydrate metabolism* (316), *Starch and sucrose metabolism* (294), and *Spliceosome* (262) (Supplementary Figure 4b).

### Development and polymorphism identification of SSR markers

A total of 108,399 primer pairs were designed in 36,133 SSR loci (Supplementary Table 5), among which 300 SSR primer pairs from different SSR loci were selected to amplify eight selected DNA samples. A total of 208 primers pairs (69.3%) successfully produced an amplicon of the expected size and 49 primer pairs (16.3%) showing good polymorphism were used to amplify the 56 *K. melanthera* accessions (Supplementary Table 6). The gel figure of Km-eSSR231 was presented in Supplementary Figure 5. A total of 358 alleles (Na) and 285.68 effective alleles (Ne) were produced, with the average values of 7.31 and 5.83, respectively (Supplementary Table 7). The average values of I and He were 0.365 and 0.464, respectively. The PIC values ranged from 0.025 to 0.431 (marker Km-eSSR42).

The gene family annotations of the transcripts where the newly developed polymorphic SSR resides were listed in Supplementary Table 6. It is noteworthy that many SSRs were annotated to genes with the important functions in plant metabolism, growth and development, and resistance to adverse growth environments. For example, seven transcripts are annotated as PK genes, five as ABC transporter genes, and some as *POT* gene, *Spt20* gene, *Hsp70* gene, *F-box* gene, and cytochrome *P450* gene.

### Population structure analysis using newly developed SSR markers

**FIGURE 6:**
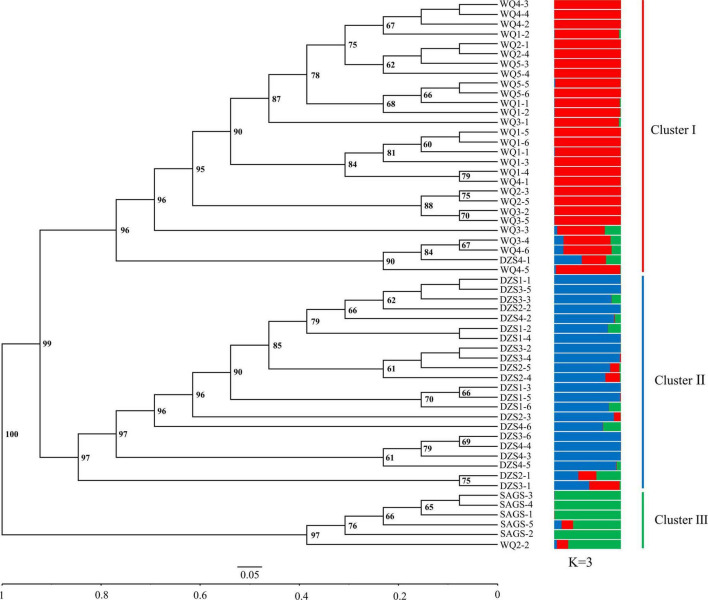
The UPGMA dendrogram and genetic structure of 56 *K. melanthera* individuals based on 49 developed SSR markers. WQ, Waqie town; DZS, Dazhasi town; SAGS, Sichuan Academy of Grassland Science.

**FIGURE 7:**
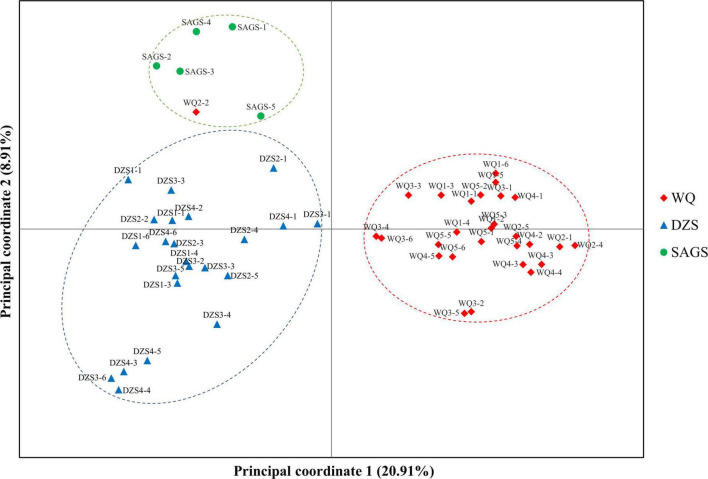
The PCoA analysis of 56 *K. melanthera* individuals based on 49 developed SSR markers.

Unweighted pair-group method with arithmetic means cluster analysis was used to construct the phylogenetic tree using 49 pairs of SSR primer developed in this study. The results showed that 56 wild *K. melanthera* germplasms could be grouped into three clusters basically corresponding to their geo-locations (Figure 6). The Cluster I included 28 germplasms collected from Waqie town (WQ) and one from Dazhasi town (DZS). The Cluster II included 22 germplasms from Dazhasi town. The Cluster III included five germplasms collected from Sichuan Academy of Grassland Sciences (SAGS) and one from Dazhasi town. STRUCTURE analysis showed that the optimal *K* value was 3 (Supplementary Figure 6), which indicated that the tested germplasms possessed three potential genetic memberships. It is interesting to note that the germplasms in each cluster had the same main genetic background based on the STRUCTURE results. The results of PCoA analysis were similar with the UPGMA (Figure 7).

### Verification of cross-species transferability

In this study, 22 newly developed SSR markers with high polymorphisms within *K. melanthera* were examined for cross-species transfer. Except for Km-eSSR193, 21 markers were successfully amplified in all four species (*K. batalinii*, *K. mutica*, *K. rigidula*, and *K. alatavica*) (Supplementary Table 8). The cluster analysis of five *Kengyilia* species was carried out based on the amplified data, and the results demonstrated that *K. alatavica* had closer genetic relationship with *K. batalinii* (Supplementary Figure 7).

## Discussion

Transcriptome sequencing can quickly and efficiently present information with wide coverage and high accuracy, which has become a good tool for the development of molecular markers and has been widely utilized in the fields of genetics and breeding, germplasm resources protection and development (Wang et al., 2018). Compared to the next-generation sequencing, full-length transcriptome sequencing can provide an efficient and convenient way to obtain transcriptome information of non-model plants without reference genomes (Wang et al., 2019; Zhao et al., 2019). Here, 96.1 GB of raw data was obtained, with the average transcripts length of 2,399 bp. Compared with other gramineous plants, it is shorter than *Saccharum officinarum* (3,099 bp) (Piriyapongsa et al., 2018) and longer than *Carex breviculmis* (2,302 bp) (Teng et al., 2019), *Cynodon dactylon* (2,317 bp) (Zhang et al., 2018), and *Lolium perenne* (2,192 bp) (Xie et al., 2020). The full-length transcriptome obtained in our study could accelerate further related studies of *K. melanthera* and its relatives.

*K. melanthera* possesses excellent resistance to drought and wind erosion, which makes it an ideal material for desertification control (Yen and Yang, 2020). However, the allohexaploid nature makes the genome assembly difficult, which limits the mining and research of excellent resistance genes contained in *K. melanthera*. Benefiting from the long-read length of third-generation transcriptome sequencing, a total of 118,341 (93.62%) non-redundant transcripts were annotated based on the public databases. Those annotated sequences of *K. melanthera* will provide a reference for the assembly of short-read transcriptome sequencing, thus laying a foundation for the subsequent exploration of drought and barren tolerance genes of *K. melanthera*. In addition to functional annotation of transcripts, CDSs, TFs, and lncRNAs were also predicted, which could provide the data reference for later related researches.

### Distribution of SSRs in transcriptome

SSRs continue to be the marker of choice for surveys of genetic diversity (Merritt et al., 2015). Compared to G-SSRs, EST-SSRs were intimately connected to the functional genes and a lot of EST-SSRs have been developed from the transcriptome data to perform the genetic diversity analysis. Our results predicted the abundant SSRs (42,433 SSRs) based on the transcriptome data of *K. melanthera*. The SSRs frequency was 35.87%, which was much higher than that of SSR frequency obtained from NGS sequencing in related *Elymus sibiricus* (8.19%, 1/6.95 kb) (Zhou et al., 2016), *Leymus chinensis* (4.38%, 1/10.78 kb) (Chen et al., 2013), and *Pennisetum purpureum* (10.89%, 1/6.45 kb) (Wang et al., 2017). The A/T and CCG/CGG rich tendency of mononucleotide and trinucleotide motifs was also consistent with the eukaryotes (Tóth et al., 2000). The most abundant dinucleotide repeat motif was AG/CT (54.75%), which was also the same as that of *E. sibiricus* (Zhou et al., 2016) and *Lolium multiflorum* (Pan et al., 2018).

Microsatellites were found to be non-randomly distributed in gene regions, including CDS, UTRs, and introns (Li et al., 2004). The results of this study showed that UTR possessed a higher number of SSRs compared to CDS regions, which was also found in other species (Vieira et al., 2016). The possible reason is that SSRs have a high mutation rate, and the structure and function of genes will be severely changed if mutation occurs in CDS regions (Xu et al., 2020). Among the six repeat types, SSRs located in the CDS region are dominated by trinucleotide repeat motifs, and their distribution proportion was much higher than that of UTRs, because trinucleotide repeat motifs are less likely to cause frameshift mutations (Metzgar et al., 2000). At the same time, 5′-UTRs contained more trinucleotide repeat motifs than 3′-UTRs, because the SSR variations in 5′-UTRs could affect gene expression (Li et al., 2004).

### Development and transferability of polymorphic SSR markers

At present, only a few RAPD (Fan et al., 2012), RAMP (Gao et al., 2016), and ISSR (Zhang et al., 2009) markers have been applied in phylogeny and genetic variation analysis of *Kengyilia*, and few reports are available on the development of SSR markers. Through transcriptome sequencing, we were able to find a large number of SSRs residing on gene sequences. In this study, 49 SSR markers with good polymorphism were selected from 300 candidate SSR markers with an average PIC value of 0.24, which was close to the *E. sibiricus* (0.25) (Hampl et al., 2001). Considering the PIC value ranged from 0 to 0.5 for SSR markers, the EST-SSR markers developed in this study possessed the potential for further genetic study of *K. melanthera* and its relatives.

It has been found that mutations in SSR motifs can affect gene regulation, transcription, and protein function (Kashi and King, 2006). The transcripts of 49 newly developed SSR markers are involved in the most important life activities of plants, such as cell signal transduction, material transport, response to abiotic stress, multiple biosynthetic pathways, and biological detoxification pathways. Given that *K. melanthera* mainly grows in high-altitude environments with insufficient water and desertification (Yen and Yang, 2020), the polymorphic SSRs located in these genes may be the result of adaptive evolution of *K. melanthera* to the environment.

Both UPGMA and PCoA analyses divided 56 *K. melanthera* accessions into three clusters, which coincided with their geographical origin, indicating that there was a high degree of genetic variation among geo-populations of *K. melanthera*. This may be due to the geographical isolation and natural selection in different populations (Kashi and King, 2006). Nevertheless, the subpopulations of wild accessions collected from Waqie and Dazhasi town were not distinguished. This is partly attributed to the high level of gene flow caused by close geographical distance between the subpopulations.

Of the 22 newly developed markers, 21 were successfully amplified in the other four *Kengyilia* species, and the transferability ratio was as high as 95%, indicating that the newly developed markers had good transferability. The SSR markers developed in this study showed a high level of transferability among the related species. This phenomenon was also found in many other species, such as *E. sibiricus* (Zhang et al., 2019), *Agropyron cristatum* (Ren et al., 2016), and *E. excelsus* (Xiong et al., 2019). In terms of geographical distribution, *K. alatavica* and *K. Batalinii* were all from Central Asia, while *K. Mutica*, *K. rigidula*, and *K. melanthera* were mainly distributed in the QTP (Yen and Yang, 2020), which was consistent with the results of clustering. Therefore, the novel markers are reliable and have wide application value in other *Kengyilia* species.

## Conclusion

Here, full-length transcriptome sequencing was performed in *K. melanthera* for the first time, and 126,410 non-redundant transcripts were annotated in multiple databases. In the absence of *K. melanthera* genome-wide information, these full-length transcriptome data will provide great help for future related research. We identified 42,433 SSR loci from the transcriptome and designed 108,399 primer pairs. In addition, the transcripts containing SSR was associated with some important biological processes. The 49 SSR markers obtained by screening showed good polymorphism, and some of the SSR markers had good transferability, which provided a basis for the genetic research in *K. melanthera* and related species.

## Data availability statement

The datasets presented in this study can be found in online repositories. The names of the repository/repositories and accession number(s) can be found below: https://www.ncbi.nlm.nih.gov/, PRJNA735213.

## Author contributions

YLX and JY conceptualized the basic idea and plan the study, and contributed to writing—reviewing and editing. YX, JMZ, and LS helped in data collection and analyses. LL, WL, and YLX performed the statistical analyses. JQZ, MHY, and DXL helped in primary draft preparation. XL, XM, and SQB contributed to supervision. All authors contributed to manuscript revision, read, and approved the submitted version.

## Footnotes

1
https://transdecoder.github.io/

## Supplementary material

Supplementary Figure 1 Images of *K. melanthera* with whole plant **(A)** and spike **(B)**.

Supplementary Figure 2 Functional classification of *K. melanthera* transcripts. The predicted functions were based on COG **(a)**, eggNOG **(b)**, and KOG **(c)**.

Supplementary Figure 3 Distributions of the major repeat types in the *K. melanthera* transcriptome.

Supplementary Figure 4 Classification of transcripts containing SSR loci based on GO **(a)** and KEGG **(b)** databases.

Supplementary Figure 5 The gel figure amplified by Km-eSSR231. The left band means 50 bp marker. The samples 20 and 43 were discarded for further analysis.

Supplementary Figure 6 Estimation of number of *K* value for 56 *K. melanthera* individuals.

Supplementary Figure 7 The UPGMA dendrogram of five *Kengyilia* species was studied based on 22 selected SSR markers.

Supplementary Table 1 *Kengyilia melanthera* accessions for SSR marker polymorphism validation and population analysis.

Supplementary Table 2 Accessions used to verify cross-species transferability of SSR markers.

Supplementary Table 3 Gene annotations of 118,341 transcripts in *K. melanthera*.

Supplementary Table 4 Transcription factor prediction in the transcriptome of *K. melanthera*.

Supplementary Table 5 Characterization of primers developed based on 36,133 SSR loci. LncRNAs were marked with red.

Supplementary Table 6 Summary of 49 selected SSR markers with high polymorphism.

Supplementary Table 7 The diversity parameters of 49 SSR markers amplified in 56 *K. melanthera* individuals.

Supplementary Table 8 The transferability of 22 selected SSR markers was validated in other four *Kengyilia* species.
