# Supplementary figures and images for: Full-length transcriptome sequencing analysis and characterization, development and validation of microsatellite markers in Kengyilia melanthera
Source: Front Plant Sci. 2022 Jul 25;13:959042. doi: 10.3389/fpls.2022.959042 (PMC9358441; doi:10.3389/fpls.2022.959042)

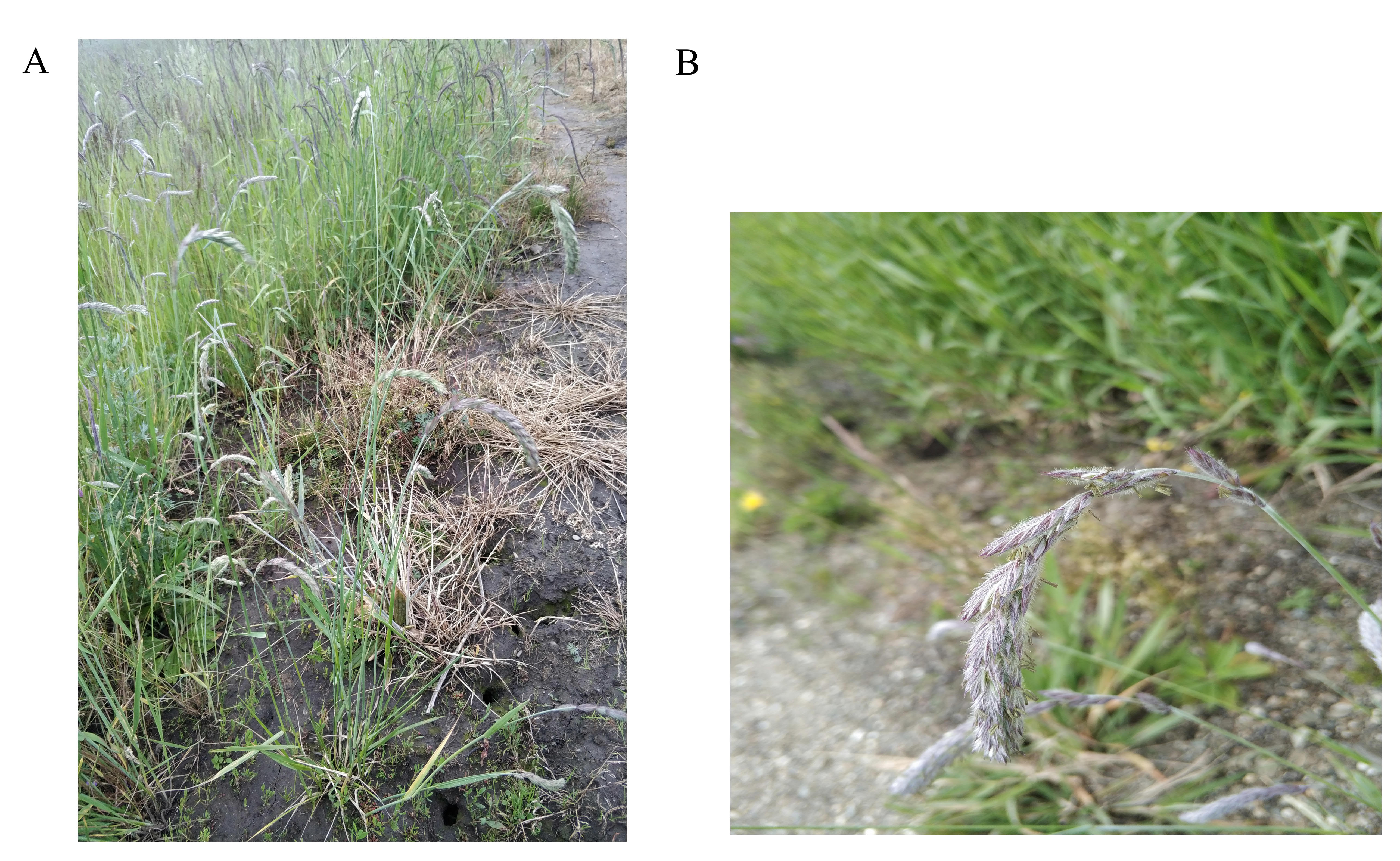

Supplement: Supplementary Figure 1 — Images of K. melanthera with whole plant (A) and spike (B). [file Data_Sheet_1.ZIP › Fig S1.jpg]

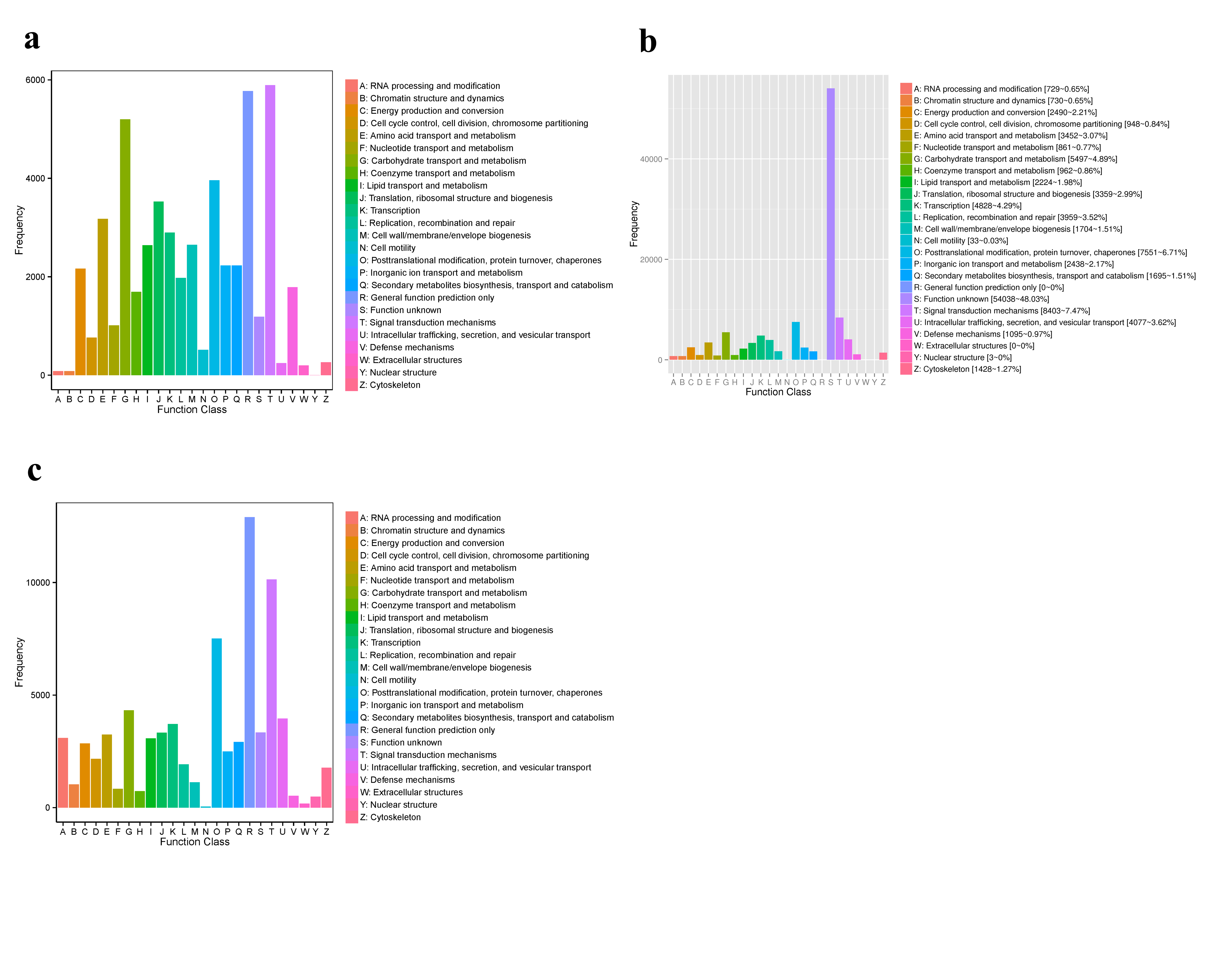

Supplement: Supplementary Figure 1 — Images of K. melanthera with whole plant (A) and spike (B). [file Data_Sheet_1.ZIP › Fig S2.tif]

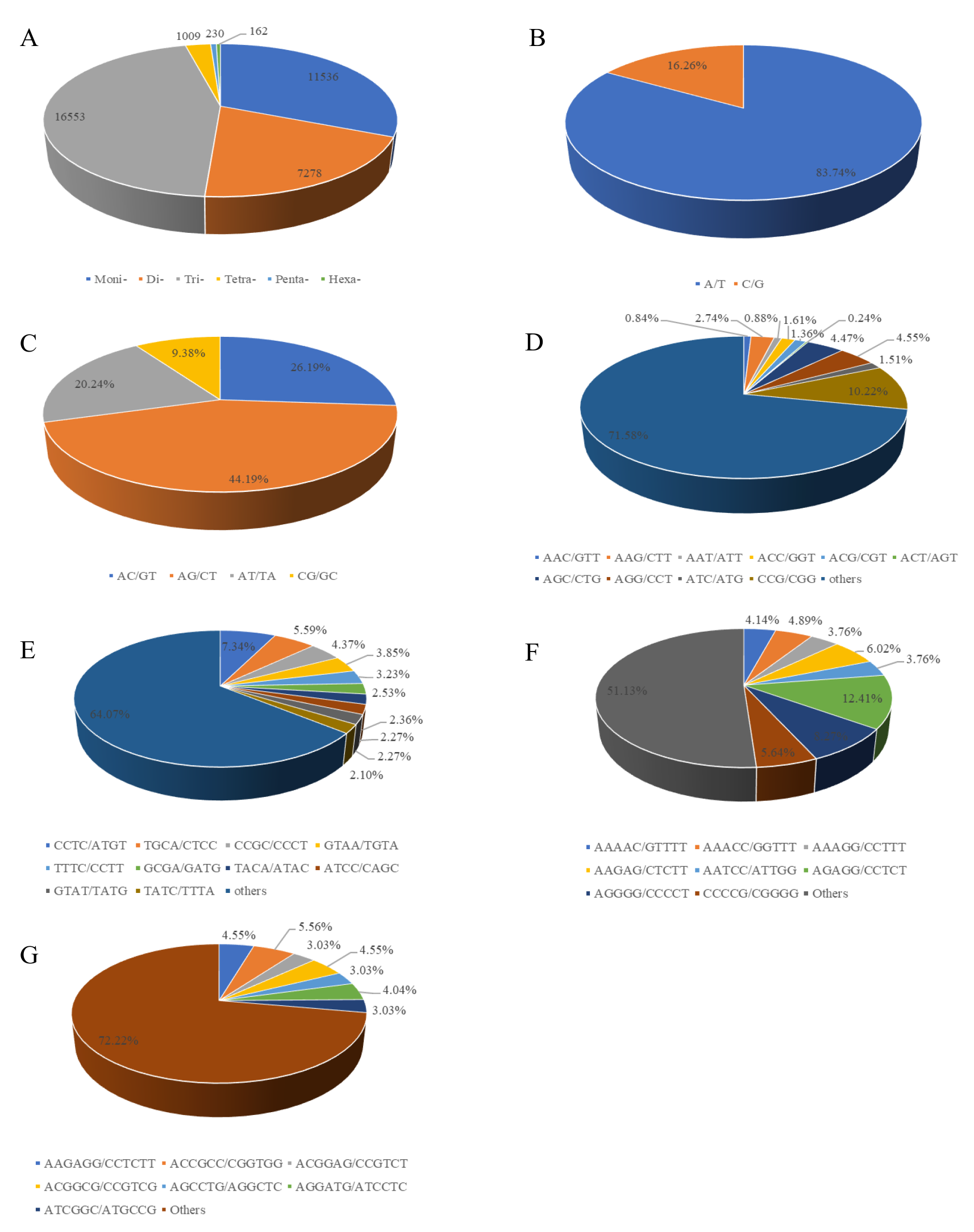

Supplement: Supplementary Figure 1 — Images of K. melanthera with whole plant (A) and spike (B). [file Data_Sheet_1.ZIP › Fig S3.tif]

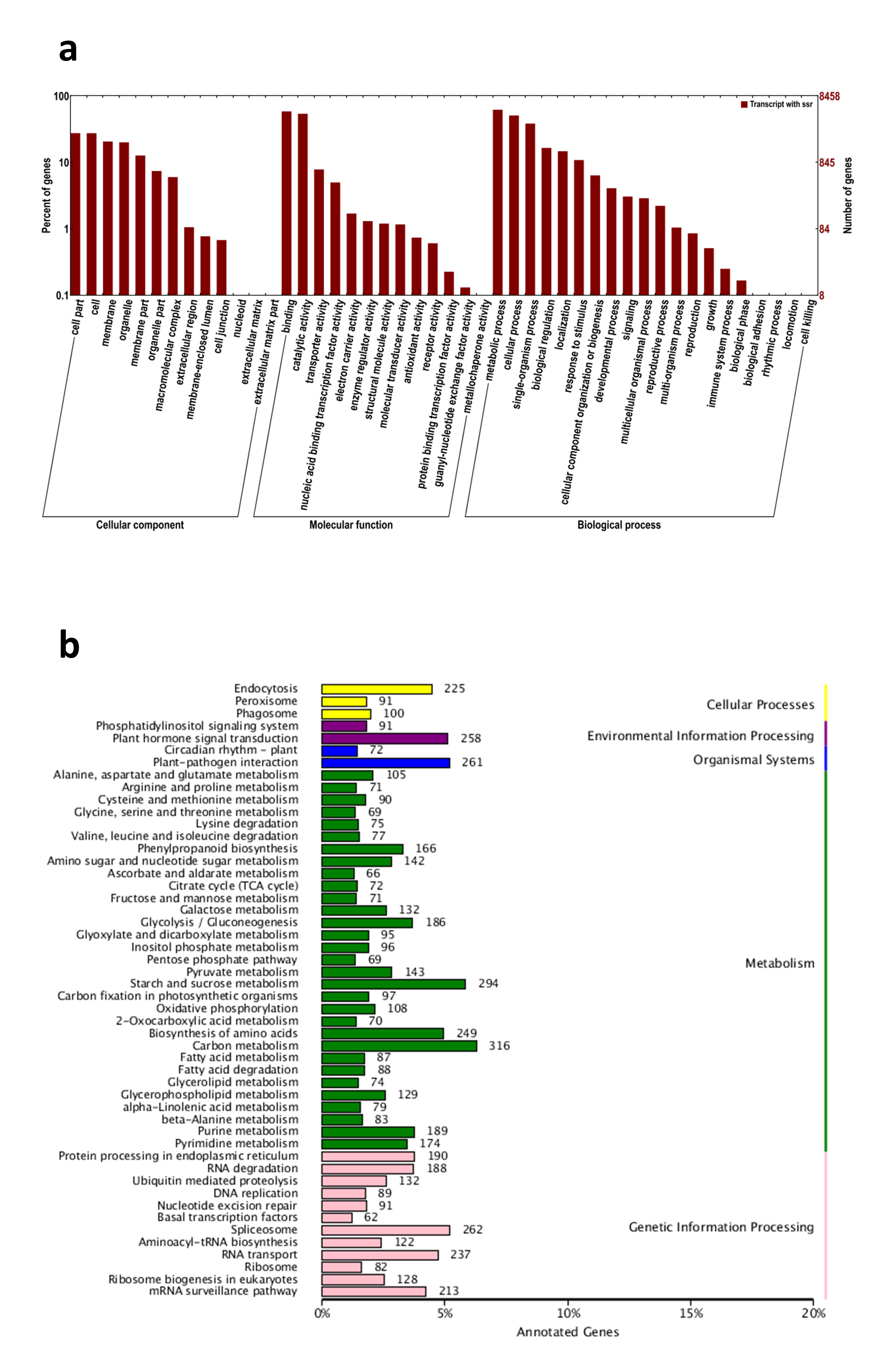

Supplement: Supplementary Figure 1 — Images of K. melanthera with whole plant (A) and spike (B). [file Data_Sheet_1.ZIP › Fig S4.png]

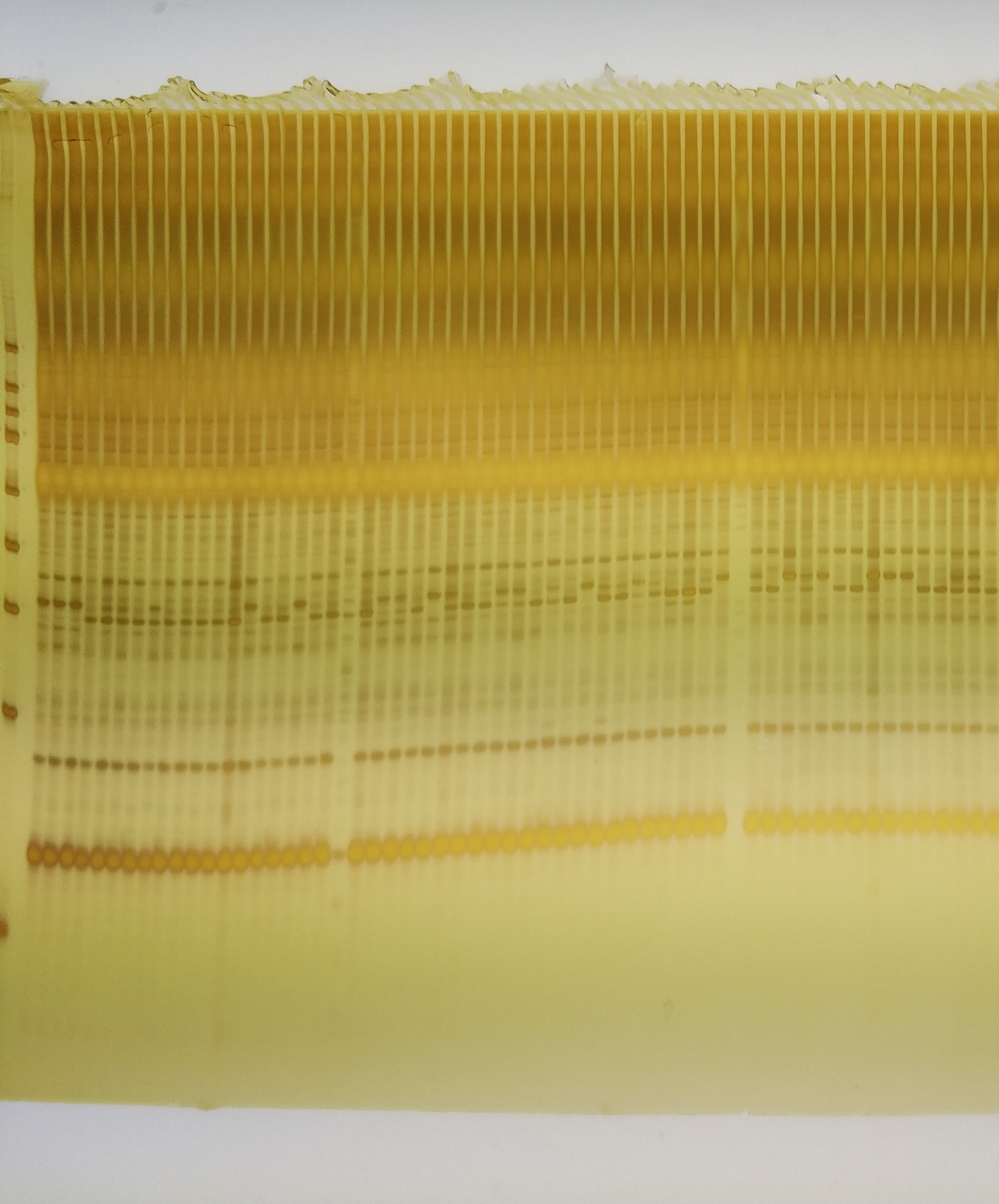

Supplement: Supplementary Figure 1 — Images of K. melanthera with whole plant (A) and spike (B). [file Data_Sheet_1.ZIP › Fig S5.jpg]

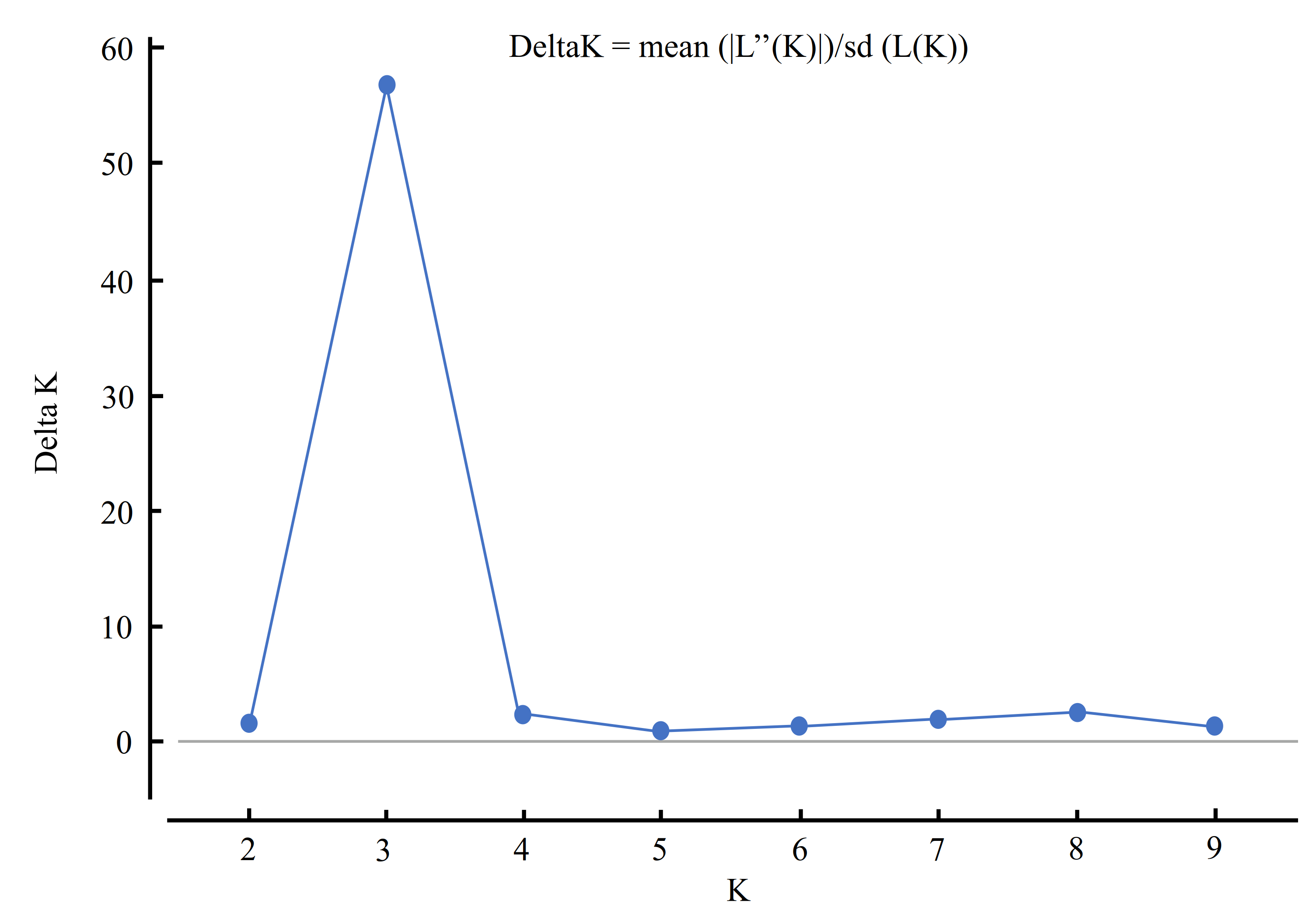

Supplement: Supplementary Figure 1 — Images of K. melanthera with whole plant (A) and spike (B). [file Data_Sheet_1.ZIP › Fig S6.tif]

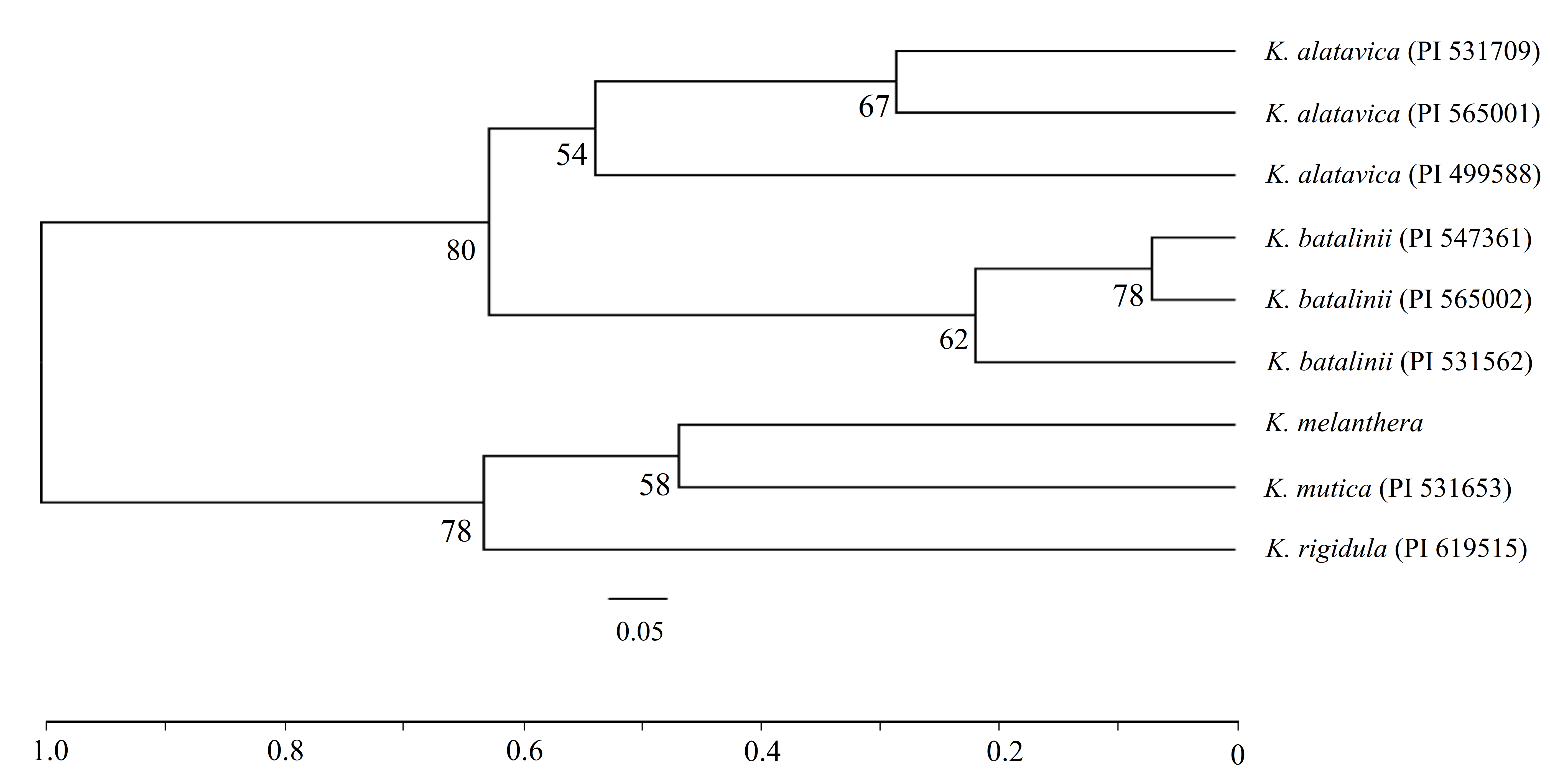

Supplement: Supplementary Figure 1 — Images of K. melanthera with whole plant (A) and spike (B). [file Data_Sheet_1.ZIP › Fig S7.tif]
